# Distribution of staphylococcal cassette chromosome *mec* types among methicillin-resistant coagulase negative staphylococci in central Iran

**Published:** 2018-02

**Authors:** Ehsanollah Ghaznavi-Rad, Nasimeh Fard-Mousavi, Ali Shahsavari, Ali Japoni-Nejad, Alex Van Belkum

**Affiliations:** 1 Molecular and Medicine Research Center, Arak University of Medical Sciences, Arak, Iran; 2 Department of Microbiology and Immunology, School of Medicine, Arak University of Medical Sciences, Arak, Iran; 3 Scientific Office, BioMérieux, Route de Port Michaud, La Balme Les Grottes 38390, France; 4 Erasmus MC, Department of Medical Microbiology and Infectious Diseases, ‘s Gravendijkwal 230, 3015 CE Rotterdam, The Netherlands

**Keywords:** Coagulase negative staphylococci, Methicillin resistance, SCC*mec* typing

## Abstract

**Background and Objectives:**

Methicillin-resistant coagulase-negative staphylococci (MR-CoNS) are important nosocomial pathogens. They may serve as a reservoir of SCC*mec*, the genomic island encoding amongst other methicillin resistance. This study was designed to determine the distribution of different SCC*mec* types from MR-CoNS isolated from clinical specimens in a tertiary hospital in central Iran, having high frequency of nosocomial methicillin-resistant staphylococcal infections.

**Materials and Methods:**

We evaluated isolates from patients attending the Vali-Asr Hospital located in the center of Iran, from February to December 2012. Multiplex PCR was performed for SCC*mec* typing. For isolates in which SCC*mec* could not be typed directly, additional *ccr* and *mec* complex analyses were performed.

**Results:**

Totally, 70 MR-CoNS isolates, comprising of 47 *S. epidermidis* strains (67%), 10 *S. saprophyticus* (14.3%), 9 *S. hemolyticus* (13%) and 4 *S. lugdunensis* (5.7%) were identified. Thirty-nine were characterized as type IVa 19 (27%), type III 11 (16%), type II 7 (10%) and type V 2 (3%). Only 20 isolates (28.6%) carried the ccr complex, while the current methods could not characterize the 11 remaining isolates.

**Conclusion:**

A high level of SCC*mec* genetic diversity was found among MR-CoNS isolates. MR-CoNS may act as a reservoir of SCC*mec* IV for MRSA. This issue should be taken into consideration seriously.

## INTRODUCTION

Coagulase-negative staphylococci (CoNS) are part of the human normal flora and are considered human opportunistic pathogens, commonly associated with severe infections such as bacteremia and septicemia, particularly in patients with indwelling devices or immunocompromised patients (1). CoNS comprise of a variety of *Staphylococcus* spp and are usually resistant to most ß-lactams antibiotics including methicillin (2). Methicillin resistance is mainly due to the expression of the *mec*A gene, which specifies a penicillin binding protein 2a (PBP2a), a transpeptidase with a low affinity for ß-lactams. *mec*A is part of SCC*mec* which often integrates at the 3’ end of a chromosomal open reading frame designated as *orf* X.

Generally, SCC*mec* contains two essential components, the mec gene complex and the ccr gene complex. The *mec* gene complex composed of *mec*A regulatory genes and it has been classified into six different classes: A, B, C1, C2, D and E. Cassette chromosome recombinase (*ccr*) genes (*ccrC* or the pair of *ccrA* and *ccrB*) encode recombinases-mediating integration and excision of SCC*mec* into and from the chromosome.

To date, 11 different types of SCC*mec* (I–XI) have been defined on the basis of the combination of *ccr* and *mec* complexes, but only type I– V are globally distributed, whilst others appear to exist as local strains in the country of origin (http://www.sccmec.org/Pages/SCC_TypesEN.html) (3).

The origin of SCC*mec* is still unknown. However, it is proposed that it was acquired by *Staphylococcus aureus* from *S. sciuri* or other *mec*A positive CoNS, especially *S. epidermidis*, which could be a potential reservoir for the SCC*mec* element, although the mechanism of inter-species transfer remains unknown.

Currently, in our hospital the incidence rate of methicillin-resistant *S. aureus* (MRSA) has increased to more than 80% in clinical isolates and the prevalence of methicillin resistant (MR) CoNS is also rising (4). The efficient management of MRSA infection in every hospital relies on a correct diagnosis, as well as understanding the antimicrobial resistance profile, epidemiology, transmission routes, appropriate therapeutics and appropriate infection control measurements. In Iran, the SCC*mec* types have been extensively studied in *S. aureus*, but little is known about MR-CoNS. Therefore, this study was designed to investigate the SCC*mec* element in CoNS isolated from clinical specimens of hospitalized patients in central Iran.

## MATERIALS AND METHODS

A total of 102 CoNS clinical isolates (December 2013 to July 2014), recovered in Vali-Asr hospital (Arak University of Medical Sciences) were subjected to this cross-sectional study.

Isolated strains were identified using conventional methods (growth on mannitol salt agar, colony morphology), Gram stain, catalase-coagulase, DNase tests, acid production from mannose, xylose, maltose, sucrose, and urease test. All the samples were checked for methicillin susceptibly by cefoxitin disk.

All the confirmed isolates were subjected to a DNA genomic extraction procedure using a commercial DNA extraction kit (Bio flux bioer, Korea) according to the manufacturer’s instruction. The presence of the *mec*A gene was confirmed in all the cefoxitin-resistant isolates, using primers published elsewhere (5).

SCC*mec* typing was performed using a multiplex PCR assay that was published previously (6). The isolates, identified by this methodology were subjected to PCR amplification of the *ccr* and *mec* complex. *ccrA1, ccrA2, ccA3* and *ccrC* primers were used to determine the *ccr* class of SCC*mec* (6). The following *mec*I, *IS1272* and *mec*C primers were used to determine the class A, B or C of *mec* complex (6, 7).

### Reference strains.

The following strains were used as references: SCC*mec* type I (NCTC 10442); type II (N315); type III (85/2082); type IVa (JCSC 4744); type IVb (JCSC 2172); type IVc (JCSC 4788); type IVd (JCSC 4469); and type V (WIS).

## RESULTS

Of the 102 isolates, 70 (68.6%) MR-CoNS belonging to four different species were identified, according to standard methods. The species distribution was as follows: *S. epidermidis* 47 (67%), *S. saprophyticus* 10 (14.3%), *S. hemolyticus* 9 (13%) and *S. lugdunensis* 4 (5.7%). These isolates were collected from different clinical specimens, including blood (n=36, 51.5%), wound (n=10, 14.3%), urine (n=17, 24%), catheter tips (n=2, 3%), drainage fluids (n=2, 3%) and sputum (n=3, 4.2%) (Table 1).

**Table 1.** Frequency of the coagulase-negative Staphylococci isolated from different clinical specimens

**Species**	***S. epidermidis***	***S. saprophyticus***	***S. hemolyticus***	***S. lugdunensis***	**Total**

**Specimen**					
Blood	30	-	4	2	36 (51.4%)
Wound	6	-	3	1	10 (14.3%)
Urine	7	10	-	-	17 (24.2%)
Catheter	1	-	1	-	2 (2.9%)
Body fluid	2	-	-	-	2 (2.9%)
Sputum	1	-	1	1	3 (4.3%)
Total	47 (67.2%)	10 (14.3%)	9 (12.9%)	4 (5.8%)	70 (100%)

All of the 70 MR-CoNS isolates contained *mec*A gene, as detected by PCR. SCC*mec* types were assigned for 39 of 70 (56%) isolates by multiplex PCR. Among these 39 isolates, different SCC*mec* types were found containing type IVa (n=19, 27%), type III (n=11, 16%), type II (n=7, 10%) and type V (n= 2, 3%). The remaining 31 isolates, in which SCC*mec* types could not be determined by multiplex PCR, were subjected to single-plex PCR assays for determination of *ccr* and *mec* complex. Of the 31 isolates, 20 were positive for *ccrA2B2* genetic element, while six of these isolates were carrying *ccrA3B3*, simultaneously. However, a further investigation through PCR assay for the determination of *mec* complex class was not successful in these isolates; therefore, despite repeated attempts *mec* complex genes could not be obtained from the remaining 11 isolates. The remaining 11 (15.7%) isolates were not typed by any of the mentioned methods (Table 2).

**Table 2.** Frequency of the different SCC*mec* types among different coagulase-negative Staphylococci

**Species**	***S. epidermidis***	***S. saprophyticus***	***S. hemolyticus***	***S. lugdonensis***

**SCC*mec***				
Iva	19	-	-	-
III	7	-	4	-
II	4	-	3	
V	-	-	-	2
*ccrA2B2*	11	3	-	-
*ccrA2B2/ccrA3B3*	4	2	-	-
Non-typeable	2	5	2	2
Total number	47 (67.2%)	10 (14.3%)	9 (12.9%)	4 (5.8%)

## DISCUSSION

In this study, the distribution of SCC*mec* types in MR-CoNS isolated from clinical specimens in a central teaching hospital in the center of Iran was evaluated. Targeting *ccr* and the *mec* gene complex through the application of different typing methods determined the SCC*mec* types in 59 of 70 isolates (6, 8).

The frequency of methicillin resistance among coagulase negative staphylococci was found to be 68.6%. This frequency rate is increasing and has reached 70%–80% in central Europe and other geographical regions since the introduction of methicillin (9, 10). For instance, the overall frequency of methicillin resistance among coagulase negative staphylococci in our neighboring country, Turkey was 83.3% (11). Methicillin-resistant *S. epidermidis* is the predominant skin flora of hospitalized patients, and colonizing methicillin-resistant *S. epidermidis* can persist on the skin for months after discharge (12). Our result is consistent with the results of other studies in the literature. However, because we have not checked the patients upon admission, we are not sure whether these isolates are acquired in the hospital or from the community. Therefore, community diffusion and silent in-hospital influx of methicillin-resistant *S. epidermidis* -SCC-*mec* IV may reflect a widely accessible reservoir of methicillin resistance for *S. aureus* in hospitals and the community (10).

Regarding SCC*mec* type distribution, our MR-CoNS isolates were found to be diverse. Up to four different types were found and 20 (28.6%) isolates only carried the *ccr* complex, while 11 isolates remained unclassified. Type IVa was the most abundant (27%), followed by types III, II and V.

Among the 47 isolates of *S. epidermidis*, 19 (40.4%), 7 (15%) and 4 (8.5%) harbor type IVa, III and II, respectively. These 11 (23.4%) only carry *ccrA2B2*, while 2 (4.3%) contain *ccrA2B2/ccrA3B3* in their cassette (13).

Several studies have found that in MRSA clone ST-239, SCC*mec* type III has been identified; this clone was found to be circulating in Iranians hospitals and other Asian countries (14–16). Hence, as it is evident that MR-CoNS is the main ecological reservoir of the SCC*mec* gene, we expected that SCC*mec* III would be the main type of MR-CoNS in the investigated isolates. To clarify this controversy, a comprehensive SCC*mec* typing of the MRSA and MR-CoNS isolated from the infection site in the hospital is needed.

Meanwhile, SCC*mec* type IV was found as a minor clone emerged in the hospital setting in different parts of Iran (17, 18). In addition, SCC*mec* IV was the most frequent SCC*mec* type (90%) isolated from CA-MRSA (community acquired –methicillin resistant *S. aureus*) in healthy carriers at central Iran (19). Varied SCC*mec* types in MR-CoNS have been distributed and are dominant in different countries. For instance, SCC*mec* type III has been found to be the most prevalent in southern Brazil (52%) (20), whereas SCC*mec* type IV has been reported to be the most common in the United Kingdom (36%) (12). In Japan, MR-CoNS strains have been steadily disseminated within the community and have become more prevalent than CA-MRSA (21). These strains predominantly harbored type IV SCC*mec* elements, similar to CA-MRSA strains disseminated throughout the world (13, 22).

Currently, the emergence of a wide range of CA-MRSA clones carrying SCC*mec* IVa is one of the most alarming issues in terms of antibiotic resistance (19, 23, 24). The small size of the type IV plays an important role in its mobility and enables easier exposure among MRSA in the community, as in the hospital (24). Because high homology has been found between *S. epidermidis* and major clones of methicillin-resistant *S. aureus* (13), it could be suggested that CA-MRSA clones could have originated from coagulase negative staphylococci (25).

Interestingly, co-colonization by methicillin susceptible *S. aureus* and methicillin-resistant *S. epidermidis*-Iva upon hospital admission, further clarify this finding (13).

The SCC*mec* structure was found to be more polymorphous in MR-CoNS, with different *ccr-mec* combinations not described in MRSA and untypable *ccr* allotypes.

In MR-CoNS strains, the presence of multiple *ccr*-carrying strains was reported; for instance, type III SCC*mec* was found to contain an SCC mercury element driven by the *ccrC3* allotype (26, 27) or type V strains, which contain *ccrAB2* and *ccrC* in community-acquired MRSA (28). This finding could be due to the addition of non-*mec*SCC elements in the SCC*mec* structure. In addition, while focusing on the composite structure of SCC*mec*, this indicates that *ccr* gene expression has an important role in the SCC*mec* exchange between species.

In addition, 20 out of 31 of the non-typable isolates (64.5%) were found to harbor the *ccrA2B2* complex that belongs to type II or IV SCC*mec.* These nontypable isolates could be similar to a few variants of SCC*mec* type II, but with lack of *kpd* operon in the J-region. Therefore, current multiplex PCR methods are not able to identify them as SCC*mec* type II (29). However, *mec* complex typing was also not able to categorize these isolates. All of these factors emphasize on a frequent loss and acquisition of mobile genetic elements in *S. epidermidis* (30).

Most of the studies have reported that methicillin-resistant *S. saprophyticus* harbor untypable SCC*mec* types (31, 32). This difficulty could be due to a combination of DCS and *mec*I amplification products that were not consistent with those known types of SCC-*mec* elements (32), or could be due to difficulties in *ccr* complex of methicillin-resistant *S. saprophyticus*, which has been previously reported (31). This is the first report to show that methicillin-resistant *S. saprophyticus* isolates from Iran carry *ccrA2B2* or *ccr-A3B3* elements. While the study of Soderquist et al. (31) found that all three non-typable *S. saprophyticus* isolates were negative for all the investigated *ccr* elements, two carried the class A *mec* gene complex. The investigation on eight *mec*A-positive *S. saprophyticus* isolates from Japan revealed that they were positive for the class A *mec* gene, but were negative for the *ccr* complex (32). It can be concluded that the recipient chromosomes of various MR-CoNS are different because of the variety in the SCC*mec* types. In addition, this finding suggests that SCC*mec* in methicillin-resistant *S. saprophyticus* should be further investigated, while a further analysis of *S. saprophyticus* prototypes is necessary.

Out of nine methicillin-resistant *S. hemolyticus* isolates, four (44.4%) were identified as type III, two (22.2%) as type II and two isolates were non-typable. *S. hemolyticus* isolates have been found to be diverse and harbor SCC*mec* II, III and IV in different countries (27, 33). Therefore, in our region, *S. hemolyticus* could be a considerable reservoir of type III and II SCC*mec*, respectively.

Interestingly, we found that two *S. lugdonensis* isolates carrying SCC*mec* V. *S. hemolyticus* isolates with type V SCC*mec* had been reported in the literature from Brazil and Taiwan and could be misidentified as MRSA (34, 35). The clinical and epidemiological significance of this resistance phenotype must be taken into consideration seriously, because of the potential danger of type V SCC*mec* in the community and in hospitals.

In conclusion, this is the first report of SCC*mec* typing among MR-CoNS isolates from Iran. A high frequency of MR-CoNS isolated from the infection site is an important finding of this study that could be correlated with a high frequency of MRSA in this hospital setting. The predominance of type IV and III SCC*mec*, which are the genetic determinants of CA and HA-MRSA, emphasize that MR-CoNS can serve as the possible sources of SCC*mec* for MRSA. The application of different typing methods was not able to determine the SCC*mec* construction of 16 (23%) isolates, indicating the need for developing or modifying a classification system for SCC*mec* types in MR-CoNS.
